# Prevalence and risk factors for cardiovascular disease among chronic kidney disease patients: results from the Chinese cohort study of chronic kidney disease (C-STRIDE)

**DOI:** 10.1186/s12882-017-0441-9

**Published:** 2017-01-14

**Authors:** Jun Yuan, Xin-Rong Zou, Si-Ping Han, Hong Cheng, Lan Wang, Jin-Wei Wang, Lu-Xia Zhang, Ming-Hui Zhao, Xiao-Qin Wang, on behalf of the C-STRIDE study group

**Affiliations:** 1 Hubei University of Chinese Medicine, Wuhan, 430065 China; 2 Renal Division, Department of Medicine, Hubei Provincial Hospital of Traditional Chinese Medicine, The Affiliated Hospital of Hubei University of Chinese Medicine, Wuhan, 430061 China; 3 Renal Division, Department of Medicine, Peking University First Hospital, Beijing, 100034 China; 4 Institute of Nephrology, Peking University, Beijing, 100034 China; 5 Key Laboratory of Renal Disease, Ministry of Health of China; Key Laboratory of Chronic Kidney Disease Prevention and Treatment, Peking University, Ministry of Education, Beijing, 100034 China

**Keywords:** Cardiovascular Disease, Cerebrovascular Disease, Chronic Kidney Disease, Cohort Study, C-STRIDE, Epidemiology, Hypertension, Risk Factors

## Abstract

**Background:**

Although a high incidence of cardiovascular disease (CVD) is observed among chronic kidney disease (CKD) patients in developed countries, limited information is available about CVD prevalence and risk factors in the Chinese CKD population. The Chinese Cohort of Chronic Kidney Disease (C-STRIDE) was established to investigate the prevalence and risk factors of CVD among Chinese CKD patients.

**Methods:**

Participants with stage 1–4 CKD (18–74 years of age) were recruited at 39 clinical centers located in 28 cities from 22 provinces of China. At entry, the socio-demographic status, medical history, anthropometric measurements and lifestyle behaviors were documented, and blood and urine samples were collected. Estimated glomerular filtration rate (eGFR) was calculated by the CKD-EPI creatinine equation. CVD diagnosis was based on patient self-report and review of medical records by trained staff. A multivariable logistic regression model was used to estimate the association between risk factors and CVD.

**Results:**

Three thousand four hundred fifty-nine Chinese patients with pre-stage 5 CKD were enrolled, and 3168 finished all required examinations and were included in the study. In total, 40.8% of the cohort was female, with a mean age of 48.21 ± 13.70 years. The prevalence of CVD was 9.8%, and in 69.1% of the CVD cases cerebrovascular disease was observed. Multivariable analysis showed that increasing age, lower eGFR, presence of hypertension, abdominal aorta calcification and diabetes were associated with comorbid CVD among CKD patients. The odds ratios and 95% confidence intervals for these risk factors were 3.78 (2.55–5.59) for age 45–64 years and 6.07 (3.89–9.47) for age ≥65 years compared with age <45 years; 2.07 (1.28–3.34) for CKD stage 3a, 1.66 (1.00–2.62) for stage 3b, and 2.74 (1.72–4.36) for stage 4 compared with stages 1 and 2; 2.57 (1.50–4.41) for hypertension, 1.82 (1.23–2.70) for abdominal aorta calcification, and 1.70 (1.30–2.23) for diabetes, respectively.

**Conclusions:**

We reported the CVD prevalence among a CKD patient cohort and found age, hypertension, diabetes, abdominal aorta calcification and lower eGFR were independently associated with higher CVD prevalence. Prospective follow-up and longitudinal evaluations of CVD risk among CKD patients are warranted.

**Electronic supplementary material:**

The online version of this article (doi:10.1186/s12882-017-0441-9) contains supplementary material, which is available to authorized users.

## Background

The prevalence of chronic kidney disease (CKD) has increased dramatically in economically developed countries as well as in developing countries. It is estimated that CKD has affected more than 100 million Chinese [1]. Many studies have showed a high incidence of cardiovascular disease (CVD) among CKD patients. The prevalence of CVD in CKD was 26.8%, 33.4%, 47.2%, and 39.1%, in CKD-ROUTE (Japan), CRIC (US), CRISIS (UK) and MERENA (Spain), respectively [2–5]. The mortality rate of end-stage renal disease (ESRD) was above 20% per year despite the use of dialysis, and more than half of the death was related to CVD [6]. Lower estimated glomerular filtration rate (eGFR) has been recognized as a strong and independent risk factor for CVD [7]. Other predictive factors contributing to higher prevalence of CVD in CKD, including hypertension, diabetes mellitus (DM), dyslipidemia, anemia (hemoglobin < 110 g/L), and albuminuria, have also been investigated substantively in epidemiological studies [2–5]. Thus, early detection and treatment of these risk factors is a key strategy in the prevention of CVD in CKD. However, little is known about the prevalence and risk factors for CVD among the Chinese population with established CKD whose genetic and economic heterogeneities are different from those in developed countries.

Therefore, we have established the Chinese cohort study of chronic kidney disease (C-STRIDE), the first national prospective CKD cohort of Chinese population. It was designed to explore risk factors for CKD progression and adverse consequences, especially CVD events. The purpose of the current study is to examine the baseline characteristics of this cohort and to identify risk factors for CVD in CKD patients.

## Methods

**Fig. 1:**
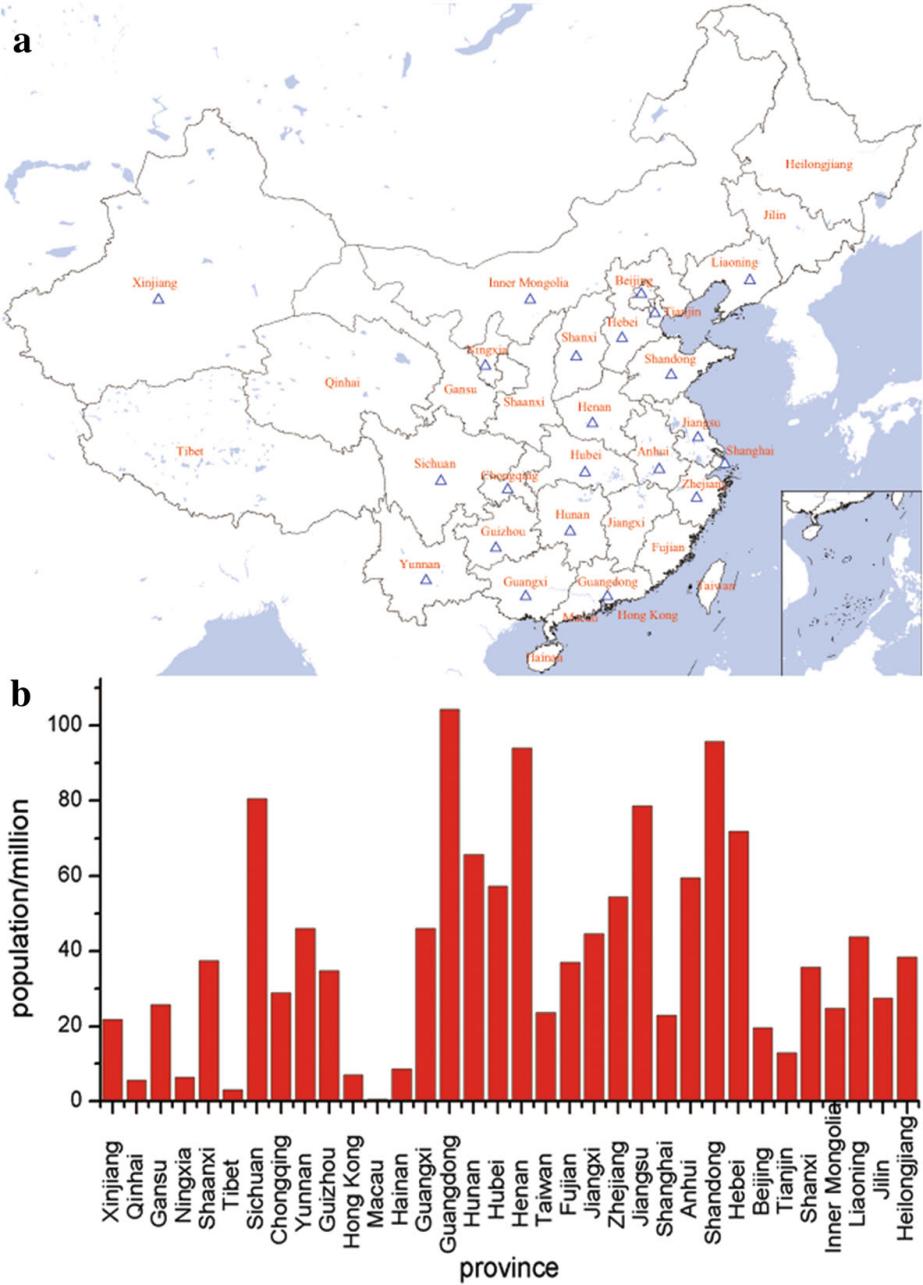
The distribution of the 39 clinical sites of the C-STRIDE Study and population size of each province in China in 2013. **a** The distribution of the clinical sites in China. The hollow triangles represent for the clinical sites in China. **b** The population size of each province in China in 2013

The design and methods of the C-STRIDE study were published in details previously [8]. The study is an ongoing multicenter prospective project involving 39 clinical centers located at 28 cities in 22 provinces of China (Fig. 1). The enrollment was carried out between November 2011 and March 2016. Altogether, 3459 Chinese patients with pre-stage 5 CKD were enrolled, and 3168 of them finished all required examinations and are included in the study.

The Renal Institute of Peking University organized the C-STRIDE study and established a steering committee consisting of nephrologists, epidemiologists and statisticians to provide training course for the research staff who performed the clinical procedures. A manual of operation procedure (MOP) was drawn up to ensure all aspects of the study were carried out in a standard and uniform manner.

CKD stages were determined by the KDIGO classification [9]. eGFR was determined with the CKD-EPI creatinine equation using serum creatinine (SCr) measured by the Roche enzymatic method [10]. For GN patients, the eGFR should be ≥15 ml/min/1.73 m^2^. For DN patients, the defining eligibility is 15 ml/min/1.73 m^2^ ≤ eGFR < 60 ml/min/1.73 m^2^ or eGFR ≥ 60 ml/min/1.73 m^2^ with “nephrotic range” proteinuria, defined as 24-h urinary protein ≥3.5 g or urinary albumin creatinine ratio (UACR) ≥2 000 mg/g. For non-GN and non-DN patients, 15 ml/min/1.73 m^2^ ≤ eGFR < 60 ml/min/1.73 m^2^ was the cutoff for enrollment.

Clinical information and biological specimens for each patient were collected at entry. Their socio-demographic status (age, gender, income, region, education), etiology of kidney disease, health history (hypertension, diabetes, and cardiovascular disease), lifestyle (smoking, exercise) and body mass index (BMI) were documented. Anthropometric measurements (weight, height, waist circumference, hip circumference, resting blood pressure, heart rate) were recorded. Electrocardiogram, abdominal aorta calcification (AAC) and 24-h urine protein were determined with standardized procedures at all centers. Biochemical parameters including SCr, calcium, phosphorus, hemoglobin (Hb), fasting glucose, hemoglobin A1C (HbA1c), triglyceride (TG), total cholesterol (TC), high density lipoprotein cholesterol (HDL-C), low density lipoprotein cholesterol (LDL-C), intact parathyroid hormone (iPTH) and high-sensitivity C-reactive protein (hs-CRP) were measured in a central laboratory to avoid testing variations among laboratories.

### Definition of hypertension, diabetes, and cardiovascular disease events

Hypertension at entry was defined as either systolic blood pressure >140 mmHg, or diastolic blood pressure >90 mmHg (confirmed by at least three elevated readings taken at least 1 week apart), or use of antihypertensive medications, or any self-reported history of hypertension. In addition, 24-hour ambulatory blood pressure was measured for every participant. Diabetes mellitus was defined as either a fasting glucose ≧7.0 mmol/L, or HbA1c ≧ 6.5%, or use of insulin or oral anti-diabetic medications, or any self-reported history of diabetes. CVD was defined as a history of myocardial infarction, hospitalization for congestive heart failure, serious cardiac arrhythmia incidents (resuscitated cardiac arrest, ventricular fibrillation, sustained ventricular tachycardia, paroxysmal ventricular tachycardia, atrial fibrillation or flutter, severe bradycardia or heart block), peripheral arterial disease (PAD), or cerebrovascular events (cerebral infarction, transient ischemic attack, cerebral hemorrhage or subarachnoid hemorrhage). Reporting of CVD was based on both the patients’ self-report and review of their medical records by trained staff on the same date of the baseline interview.

### Statistical analysis

The statistical analysis for C-STRIDE has been previously described [8]. Baseline values are presented as mean ± standard deviation (SD) or medians and interquartile ranges for continuous variables, and as numbers and percentages for categorical data. Baseline characteristics were compared between groups using analysis of variance (ANOVA), or chi-square tests, as appropriate. If the distribution of the continuous variable did not satisfy normal distribution, the Kruskal-Wallis rank sum test was used. The cardiovascular risk factors were analyzed with covariates with multivariable logistic regression models. The crude and multivariable adjusted odds ratios (aOR) with 95% confidence interval (CI) are presented. Covariates included in the multivariable logistic regression models were gender, age (18–44 (as reference) vs 45–64 vs 65–74), smoking history (yes or no), exercises more than 3.5 h per week (yes or no), hypertension (yes or no), SBP > 130 mmHg (yes or no), diabetes (yes or no), BMI≧24.0 kg/m^2^ (yes or no), CKD stages (stage 1–2 (as reference) vs 3a vs 3b vs 4), Hb < 11 g/dl (yes or no), serum calcium <8.4 mg/dl (yes or no), serum phosphorus > 4.5 mg/dl (yes or no), iPTH > 65 pg/ml (yes or no), LDL-C > 120 mg/dl (yes or no), HDL-C < 35 mg/dl (yes or no), TG > 150 mg/dl (yes or no), AAC (yes or no).

All *P* values are two-sided, and *P* < 0.05 was considered statistically significant. Analyses were conducted with SAS software (version 9.4).

## Results

### Baseline demographic and clinical characteristics

Anticipated and actual target distributions of CKD etiology and renal function are shown in Additional file 1: Table S1. The actual percentage of participants with glomerulonephritis (GN) was 60.6%, two times higher than the targeted 30%. The percentages of diabetic nephropathy (DN) and other causes were 13.9% and 25.6%, much lower than the anticipated 30% and 40%, respectively. Other causes include hypertensive renal damage, chronic pyelonephritis, hyperuricemic nephropathy, tubulointerstitial lesion and obstructive nephropathy. The proportions of participants with eGFR (ml/min/1.73 m^2^) < 45 and ≥45 were 53.5% and 46.5%, consistent with the target of 40–60%. The proportions of participants in CKD stage 1 and 2, stage 3a, stage 3b and stage 4 were 30.8%, 15.7%, 24.3%, and 29.3%, respectively.

The baseline demographic characteristics of the cohort are shown in Table 1. The final enrolled cohort had a mean age of 48.21 ± 13.7 years with 40.8% of women. Totally, 56.0% of the enrollments completed a high school education, and 36.1% had annual income ≦RMB 30,000 Yuan. The 2015 per capita disposable income of urban residents in China is RMB 31,195 Yuan “(http://www.stats.gov.cn/tjsj/zxfb/201602/t20160229_1323991.html)”. The cohort is regionally diverse with 916 (28.9%) subjects from south of Yellow River and 2252 (71.1%) patients from the north. Mean BMI was 24.47 kg/m^2^, with 53.4% of all participants having a BMI ≧24 kg/m^2^. 38.2% of the cohort participants were current smokers, and almost half of the participants exercised less than 3.5 h per week. Table 1 indicated that the CKD participants with CVD were more likely to be older, male, from the north, current smokers, and higher BMI than those without CVD (*P* < 0.005).

**Table 1:** Baseline demographic characteristics of participants of C-STRIDE Study (Nov 2011–Mar 2016)

Variable	CVD				*P*
	Total	Yes	No	Missing value	
	(*n* = 3168)	(*n* = 311)	(*n* = 2857)		
Age (yr)	48.21 ± 13.70	58.59 ± 10.51	47.08 ± 13.53	0	<0.001
Gender				0	
Male	1876 (59.22)	215 (69.13)	1661 (58.14)		<0.001
Female	1292 (40.78)	96 (30.87)	1196 (41.86)		
Annual income				139	
≤ 30 000 yuan	1092 (36.05)	119 (39.53)	973 (35.67)		0.18
> 30 000 yuan	1937 (63.95)	182 (60.47)	1755 (64.33)		
Educational attainment				22	
Junior high school degree or below	1384 (43.99)	150 (48.39)	1234 (43.51)		0.10
High school degree or above	1762 (56.01)	160 (51.61)	1602 (56.49)		
Region				0	
South	916 (28.91)	60 (19.29)	856 (29.96)		<0.001
North	2252 (71.09)	251 (80.71)	2001 (70.04)		
BMI (kg/m^2^)	24.47 ± 3.63	25.04 ± 3.35	24.41 ± 3.65	242	<0.001
BMI category (kg/m^2^)				242	
< 24	1364 (46.62)	96 (35.29)	1268 (47.78)		<0.001
≥ 24	1562 (53.38)	176 (64.71)	1386 (52.22)		
Tobacco use				62	
Yes	1185 (38.15)	156 (50.98)	1029 (36.75)		<0.001
Exercise (hours/week)				758	
≥ 3.5	1239 (51.41)	124 (52.99)	1115 (51.24)		0.61
< 3.5	1171 (48.59)	110 (47.01)	1061 (48.76)		
eGFR (ml/min/1.73 m^2^)	50.72 ± 30.03	36.71 ± 18.87	52.25 ± 30.62	0	<0.001

Continuous variables are presented as mean ± SD. Categorical data are presented as numbers (n) of patients and percentages. *BMI* body mass index

### Baseline CVD prevalence in different stages of CKD

The baseline CVD prevalence in different stage of CKD is shown in Table 2. The overall CVD prevalence of the cohort was 9.8%, in which the percentages of MI, CHF, cerebrovascular disease and PAD were 20.6%,9.0%,69.1% and 16.1%, respectively. The prevalence of cerebrovascular events was significantly higher than that of other cardiovascular events. The participants with advanced CKD were more likely to have CVD. The prevalence of MI increased with declining eGFR, with percentage of 0.6, 1.8, 2.9, 2.9%, respectively (*P* for trend = 0.001). The same pattern was observed with cerebrovascular disease (*P* for trend < 0.001) and PAD (*P* for trend = 0.001). The proportions of MI, cerebrovascular disease and PAD were significant higher in CKD stages 3b and 4 (eGFR < 45 ml/min/1.73 m^2^) (*P* < 0.001). The proportion of CHF presented a gradual increment with CKD progression, but no significant difference was observed through eGFR groups (*P* for trend = 0.14).

**Table 2:** Baseline prevalence rate of CVD in different stages of CKD in C-STRIDE Study (Nov 2011–Mar 2016)

Variable		eGFR (ml/min/1.73 m^2^)				*P* for trend
	Total	>60	45–60	30–45	15–30	
	(*n* = 3168)	(*n* = 975)	(*n* = 497)	(*n* = 769)	(*n* = 927)	
MI	64 (20.58)	6 (0.62)	9 (1.81)	22 (2.86)	27 (2.91)	0.001
CHF	28 (9.00)	3 (0.31)	5 (1.01)	9 (1.17)	11 (1.19)	0.14
Cerebrovascular disease	215 (69.13)	22 (2.26)	44 (8.85)	58 (7.54)	91 (9.82)	<0.001
PAD	50 (10.08)	3 (0.31)	8 (1.61)	13 (1.69)	26 (2.81)	<0.001
Total CVD	311 (9.82)	31 (3.18)	59 (11.87)	86 (11.18)	135 (14.56)	

Categorical data are presented as numbers (n) of patients and percentages. *MI* myocardial infarction, *CHF* congestive heart failure, *PAD* peripheral arterial disease

### Traditional CVD risk factors

Table 3 shows the baseline characteristics of the traditional risk factors for CVD. Comparisons between patients with and without CVD are presented. The participants with CVD were more likely to have hypertension and diabetes (*P* < 0.001). SBP, blood glucose and HbA1C were significantly higher in CKD participants with CVD than without CVD (*P* < 0.001). The TC, LDL-C and HDL-C were also different with and without CVD (*P* < 0.05). However, no significant difference was observed in DBP (*P* = 0.83) or TG (*P* = 0.72).

**Table 3:** Baseline characteristics of traditional risk factors characteristics for CVD in C-STRIDE Study (Nov 2011–Mar 2016)

Variable	CVD				*P*
	Total	Yes	No	Missing value	
	(*n* = 3168)	(*n* = 311)	(*n* = 2857)		
Hypertension	2106 (77.80)	232 (93.55)	1874 (76.21)	461	<0.001
SBP (mmHg)	129.29 ± 17.51	134.71 ± 17.25	128.74 ± 17.44	342	<0.001
DBP (mmHg)	80.93 ± 11.65	80.48 ± 10.98	80.98 ± 11.71	342	0.83
Diabetes	697 (22.27)	135 (43.41)	562 (19.94)	38	<0.001
Blood glucose (mg/dl)	94.32 ± 28.62	103.86 ± 30.78	93.24 ± 27	221	<0.001
G-Hb (%)	5.92 ± 1.24	6.58 ± 1.56	5.84 ± 1.17	1625	<0.001
TC (mg/dl)	231.63 ± 496.52	194.51 ± 95.90	235.89 ± 522.81	192	0.02
LDL-C (mg/dl)	108.66 ± 103.64	99.38 ± 39.06	109.82 ± 108.66	242	0.002
HDL-C (mg/dl)	44.86 ± 43.70	41.38 ± 13.92	45.24 ± 46.02	241	0.001
TG (mg/dl)	255.90 ± 1125.41	193.03 ± 128.39	262.98 ± 1185.9	193	0.72

Continuous variables are presented as mean ± SD. Categorical data are presented as numbers (n) of patients and percentages. *SBP* systolic blood pressure, *DBP* diastolic blood pressure, *G*-*Hb* glycosylated hemoglobin, *TC* total cholesterol, *LDL*-*C* low density lipoprotein cholesterol, *HDL*-*C* high density lipoprotein cholesterol, *TG* triglycerides

Lower lipid levels were observed in the CVD-CKD population compared to the non-CVD CKD population (P < 0.001). The CVD population likely attracts more attention for hyperlipidemia and receives prescription medications for lowering lipid levels, whereas the non-CVD population is less likely to receive treatment. This is confirmed by our finding that the proportion of statin treatment was 37.9% in the CVD patients versus 17.0% in the non-CVD patients.

### Non-traditional CVD risk factors

Table 4 shows the baseline characteristics of non-traditional risk factors for CVD. The participants with CVD had higher SCr than those without CVD (*P* < 0.001). iPTH and abdominal aorta calcification were significantly different with and without CVD as well (*P* < 0.001). Significant difference was also found in hemoglobin and Hs-CRP (*P* < 0.05). There were no significant differences in UTP/24 h, serum calcium and phosphorus.

**Table 4:** Baseline characteristics of non-traditional risk factors characteristics for CVD in C-STRIDE Study (Nov 2011–Mar 2016)

Variable	CVD			Missing value	*P*
	Total	Yes	No		
	(*n* = 3168)	(*n* = 311)	(*n* = 2857)		
eGFR (ml/min/1.73 m^2^)	50.72 ± 30.03	36.71 ± 18.87	52.25 ± 30.62	0	<0.001
Urine protein/24 h (g)	0.94 (0.34,2.30)	1.04 (0.24,2.84)	0.93 (0.35,2.26)	438	0.53
Serum calcium (mg/dl)	8.92 ± 0.76	9.00 ± 0.76	8.92 ± 0.76	160	0.34
Serum phosphorus (mg/dl)	3.66 (3.22,4.12)	3.66 (3.22,4.19)	3.66 (3.22,4.12)	163	0.55
Total iPTH (pg/mL)	46.66 (29.6,74.61)	55.85 (37.66,91.32)	45.79 (28.83,71.94)	578	<0.001
AAC (n)	2.62 ± 4.02	3.51 ± 4.25	2.52 ± 3.98	0	<0.001
Hemoglobin (g/dl)	12.76 ± 2.26	12.38 ± 2.16	12.80 ± 2.27	268	0.002
Hs-CRP (mg/L)	1.32 (0.53,3.20)	1.71 (0.64,4.46)	1.28 (0.51,3.10)	504	0.004

Continuous variables are presented as mean ± SD, or median with interquartile ranges. Categorical data are presented as numbers (n) of patients. *SCr* serum creatinine, *AAC* abdominal aorta calcification, *hs*-*CRP* high-sensitivity C-reactive protein

### Overall CVD risk factors

The results of multiple logistic regression analysis of the traditional and non-traditional risk factors for CVD prevalence at enrollment are shown in Table 5. ORs were adjusted mutually for all potential risk factors listed in the table. In multivariable analysis, the variables significantly associated with the presence of CVD were age, hypertension, diabetes mellitus, CKD stage, and AAC. The risk factors of CVD with higher ORs were older age (OR: 3.78; 95% CI: 2.55–5.59) (*P* < 0.001) in age 45–64 years, (OR: 6.07; 95% CI: 3.89–9.47) (*P* < 0.001) in age 65–74 years), followed by lower eGFR (OR: 2.07;95% CI:1.28–3.34) in CKD stage 3a (*P* = 0.003), (OR: 1.66; 95% CI: 1.00–2.62) in CKD stage 3b (*P* = 0.032), (OR: 2.73; 95% CI: 1.72–4.36) in CKD stage 4 (*P* < 0.001)), hypertension (OR: 2.57; 95% CI:1.50–4.41) (*P* < 0.001), AAC (OR: 1.82; 95% CI: 1.23–2.70) (*P* = 0.003) and diabetes (OR: 1.70; 95%CI:1.30–2.23) (*P* < 0.001).

**Table 5:** Risk factors for the prevalence of CVD in C-STRIDE Study (Nov 2011 - Mar 2016)

	Univariate OR (95% CI)	*P*	Age and sex adjusted OR (95% CI)	*P*	Multivariate adjusted OR (95% CI)^a^	*P*
Gender						
Male	1.61 (1.25–2.07)	<0.001	−	−	1.36 (0.96─1.94)	0.09
Female (ref)	1		−		1	
Age						
18–44 (ref)	1		−		1	
45–64	5.16 (3.56–7.48)	<0.001	−	−	3.78 (2.55–5.59)	<0.001
65–74	10.25 (6.85–15.34)	<0.001	−	−	6.07 (3.89–9.47)	<0.001
Tobacco use (yes/no)	1.79 (1.41–2.27)	<0.001	1.46 (1.071–1.99)	0.017	1.31 (0.95–1.81)	0.10
Exercises < 3.5 h/week (yes/no)	1.07 (0.82–1.41)	0.61	0.81 (0.61–1.07)	0.14	0.80 (0.60–1.08)	0.14
Diabetic (yes/no)	3.08 (2.42–3.93)	<0.001	1.93 (1.49–2.49)	<.0001	1.70 (1.30–2.23)	<0.001
Hypertension (yes/no)	4.53 (2.70–7.58)	<0.001	3.37 (2.00–5.68)	<.0001	2.57 (1.50–4.41)	<0.001
HDL-C <35 mg/dl	1.40 (1.08–1.81)	0.01	1.26 (0.96–1.65)	0.09	1.14 (0.84–1.54)	0.41
LDL-C >120 mg/dl	0.76 (0.58–1.01)	0.05	0.74 (0.56–0.99)	0.04	0.81 (0.59–1.10)	0.17
TG >150 mg/dl	1.06 (0.84–1.35)	0.63	1.09 (0.85–1.40)	0.48	0.98 (0.75–1.28)	0.87
BMI						
<24 (ref)	1		1		1	
≥24	1.68 (1.29–2.18)	<0.001	1.39 (1.06–1.81)	0.0174	1.30 (0.97–1.72)	0.08
CKD stages						
1–2(ref)	1		1		1	
3a	4.10 (2.62–6.43)	<0.001	2.60 (1.64–4.13)	<.0001	2.07 (1.28–3.34)	<0.003
3b	3.83 (2.51–5.85)	<0.001	2.22 (1.44–3.44)	0.0003	1.66 (1.00–2.62)	0.03
4	5.19 (3.47–7.76)	<0.001	3.28 (2.16–4.97)	<.0001	2.73 (1.72–4.36)	<0.001
P >5 mg/dl	1.03 (0.72–1.45)	0.89	1.25 (0.87–1.795)	0.23	0.96 (0.66–1.42)	0.85
Ca <8.4 mg/dl	1 (0.74–1.35)	0.10	0.97 (0.71–1.33)	0.86	0.97 (0.69–1.36)	0.85
IPTH >65 pg/mL	1.62 (1.25–2.09)	<0.001	1.42 (1.09–1.85)	0.01	1.03 (0.77–1.40)	0.83
AAC	3.71 (2.58–5.33)	<0.001	2.18 (1.498–3.18)	<.0001	1.82 (1.23–2.70)	0.003
Hb <11 g/dl	1.06 (0.78–1.43)	0.72	0.93 (0.68–1.27)	0.64	0.67 (0.47–0.95)	0.03

Note: ^a^All variables listed in the table were included in the multivariate adjusted analysis. *OR* odds ratio, *CI* confidence interval, *LDL*-*C* low density lipoprotein cholesterol, *HDL*-*C* high density lipoprotein cholesterol, *TG* triglycerides, *BMI* body mass index, *P* serum phosphorus, *Ca* serum calcium, *iPTH* intact parathyroid hormone, *AAC* abdominal aorta calcification, *Hb* hemoglobin

## Discussion

C-STRIDE is a prospective observational multicenter study of the risk factors for CVD in stage 1–4 CKD. Here we investigated the prevalence and risk factors of CVD in CKD populations. We report that the overall prevalence of CVD among 3168 participants was 9.8% at enrollment. The percentage of different CVD subtypes among the subset of patients with CVD was MI 20.6%, CHF 9.0%, cerebrovascular disease 69.1%, and PAD 10.1%, respectively. Our results also show that age, diabetes, hypertension, abdominal aorta calcification and stage 3 & 4 CKD are significantly associated with the prevalence of CVD.

C-STRIDE was designed to establish a Chinese cohort similar to the CRIC study [11], and to examine risk factors for CKD progression and CVD development in CKD patients with an eGFR between 15–90 ml/min/1.73 m^2^. C-STRIDE’s cohort consists of Chinese living in China, while CRIC is a mix of 45% White, 46% Black, and 5% Hispanic participants living in the US. There are many differences between Chinese and Western populations, such as ethnicity, calorie intake, and body size [12]. These differences are apparent between the C-STRIDE and CRIC cohorts, which also show differences in age, causes of CKD, prevalence of hypertension, diabetes and CVD, BMI, and eGFR. Any of these differences could affect the progression and treatment of CKD. As shown in Table 6, the C-STRIDE participants were younger with a lower average BMI, and with a lower prevalence of diabetes, hypertension and CVD. The C-STRIDE baseline indicated that age is an independent and graded risk factor for CVD events in 45–74 year old patients. China is a rapidly aging society in which more than one quarter of Chinese will be older than 65 years by 2050 [13]. The C-STRIDE study will help clarify the dimension of risks for ESRD and CVD among aging individuals with CKD. As summarized in Tables 3, 4 and 5, the C-STRIDE cohort exhibits numerous risk factors for CVD and several differences with the CRIC cohort. The baseline prevalence of CVD was 33.4% in CRIC, more than three times the 9.8% prevalence reported in C-STRIDE. The blood glucose control in diabetic participants was also better in C-STRIDE (mean A1C 6.0%) versus CRIC (mean A1C 7.7%). Finally, the mean BMI in C-STRIDE was 24.47 kg/m^2^, considerably lower than that in CRIC (32.1 kg/m^2^). A comparison of baseline characteristics between multiple CKD cohort studies is shown in Table 6 [2–5].

**Table 6:** Comparison of baseline characteristics of CKD cohort studies

	C-STRIDE China *n* = 3168	ROUTE Japan *n* = 1138	CRIC US *n* = 3612	CRISIS UK *n* = 1325	MERENA Spain *n* = 1129
Inclusion range of eGFR (ml/min/1.73 m^2^)	15–90	0–90	20–70	10–60	15–60
Age (years)	48.2	68	58.2	65.1	68
Male gender (%)	59.2	69.6	54	63.7	64
BMI (kg/m^2^)	24.5	23	32.1		28.4
Actual eGFR (ml/min/1.73 m^2^)	50.7	32.7	43.4	30.9	28
Diabetes (%)	21.7	37.1	47	32.4	40.8
Hypertension (%)	66.5	90.2	86		92.7
SBP (mmHg)	129.3	140	127.7	138.3	141
DBP (mmHg)	80.9	78	71.4	75.2	76
Hb (g/dl)	12.76	11.9	12.7	12.41	12.8
HDL-C (mg/dl)	44.86				
LDL-C (mg/dl)	108.66	110	102.5		116
Ca (mg/dl)	8.9	9.1	9.2	9.14	
P (mg/dl)	3.7	3.6	3.7	3.72	3.7
iPTH (pg/ml)	63.6	109	53	93.2	145
Proteinuria (mean)		2.16 g/24gCr		1.08 g/24 h	1.2 g/24 h
(median)	0.94 g/24 h	0.74 g/gCr	0.17 g/24 h		
CVD prevalence (%)	9.8	26.8	33.44	47.2	39.1

*eGFR* estimated glomerular filtration rate, *BMI* body mass index, *SBP* systolic blood pressure, *DBP* diastolic blood pressure, *Hb* hemoglobin, *HDL*-*C* high density lipoprotein cholesterol, *LDL*-*C* low density lipoprotein cholesterol, *Ca* calcium, *P*, phosphorus, *iPTH* intact parathyroid hormone, *CVD* cardiovascular disease, *g*/*gCr* gram per gram creatinine

The overall CVD prevalence of 9.8% in CKD patients is much lower than reported in developed countries including Japan, but much higher than the overall percentage of 1.4% in the general Chinese population [14]. The significantly lower prevalence of baseline CVD observed in our study compared to similar cohorts might be attributable to the higher average eGFR, lower prevalence of diabetes and hypertension, and/or the younger age of subjects. These variables have been confirmed to be independent risk factors for CVD among CKD patients [15–19]. Deserving additional attention is the prominence of cerebrovascular disease among C-STRIDE participants exhibiting CVD. This is similar to the findings of the ROUTE study (Japan) [20], but different from the MERENA (Spain) [5] and CRIC (USA) [21] studies, in which heart disease and PAD constituted the majority of CVD events. There are two possible explanations for the high incidence of cerebrovascular disease. First, the C-STRIDE study excluded CKD patients with NYHA Class III or IV heart failure. Second, it appears that the Chinese general population may have a higher CVA prevalence than is observed in other countries. In a Chinese cohort study of ischemic cardiovascular disease, 45 cases (5.4%) of ischemic stroke and 24 cases (2.9%) of coronary heart disease were reported in 840 middle age men followed for 20 years [22]. The Japan Public Health Center-based prospective Study revealed 1,565 strokes (2.7%) among 57,017 subjects in a Japanese population-based cohort [23].

Based on the results of previous similar cohorts including the US CRIC [11] and the Japan CKD-JAC [24] studies, we had anticipated that the distribution of CKD etiology in C-STRIDE would be 30% glomerulonephritis (GN) and 30% diabetic nephropathy (DN) [8]. However, the actual distribution of CKD etiology was GN 60.6% (twice as high as the targeted 30%), DN 13.9% (less than half of the targeted 30%) and other causes 25.6%. This is consistent with the data from the Chinese Renal Data System, a national registry system for patients undergoing dialysis, which revealed that in China glomerular disease was the most common cause of ESRD (57.4%), followed by DN (16.4%), hypertension (10.5%), and cystic kidney disease (3.5%) [25]. Together, these data indicate that the etiological constituents of CKD in China are different from those reported in developed countries, where the leading cause of ESRD is DN [2–5]. Nevertheless, China has the highest overall number of diabetic patients in the world, rising rapidly from 92.4 million in 2007 to 113.9 million diabetic patients in 2013 [26]. Therefore, diabetes complications such as DN will likely become the main cause of ESRD in the coming decades.

Proteinuria is considered a risk factor for CVD and mortality in patients with CKD. Microalbuminuria, or even normal-range albuminuria, constitutes a risk for CVD [27–32]. For instance, the AASK study of African Americans, which investigated the cardiovascular and renal outcomes of 59,508 participants with stage 1–3 CKD, indicated a significantly increased risk of CVD with higher urinary albumin excretion, despite relatively low levels of baseline proteinuria [31]. Likewise, in a population-based cohort study in Taiwan, elevated albuminuria was a key predictor of progression to CKD or ESRD as well as indicating a higher risk of CVD and mortality [32]. The amount of urinary protein in the C-STRIDE patients (0.94 g/24 h) was higher compared with the CRIC cohort (0.17 g/24 h). In the Chinese cohort, urine protein was not significantly associated with CVD in CKD (*P* = 0.526) (Table 4). This is different from the results found in Japan [20] and US [21], where increased proteinuria was associated with a higher CVD prevalence.

It is generally thought that albuminuria always precedes loss of renal function in diabetic kidney disease [33]. However, an increasing number of studies have cast doubt on this classic paradigm. In a large number of recent studies, 20–39% of patients with diabetes and reduced eGFR had normal albuminuria [34–37]. In some clinical trials [38, 39], improvement in proteinuria did not translate into increased GFR or reduced end points such as the need for dialysis or death. Therefore, the role of proteinuria in representing renal function and in predicting adverse outcomes of CKD warrants further research. At baseline of our study, proteinuria was not associated with CVD. Long-term follow-up will provide more information to help answer this question.

Over the past two decades the leading causes of mortality and morbidity have shifted from infectious diseases to non-communicable disease such as vascular disease, renal disease and DM. These disorders have become major public health problems in developed and developing countries alike, imposing heavy economic burdens [40, 41]. The relationship between DM and CVD has been demonstrated in a series of studies [2–5, 42]. One recent study reported that the prevalence of DM among a representative sample of Chinese adults was 11.6%, and the prevalence of pre-diabetes was 50.1% [26]. These statistics illustrate the importance of DM as a public health problem in China and suggest that DM will become the leading future cause of ESRD in China [43]. Indeed, DN now accounts for 46.2% and 43.2% of ESRD cases in economically advanced regions such as Hong Kong and Taiwan [25]. Unfortunately, despite recent improvements in glycemic and blood pressure control as well as proteinuria reduction, DN remains the leading cause of ESRD in developed countries [44]. Therefore, there is an urgent need for development of novel therapeutic approaches that offer effective nephroprotection and that block key pathogenic pathways leading to diabetic kidney disease.

Hypertension is a main cause of secondary CKD in China [45]. Numerous studies have demonstrated hypertension as an important risk factor for CVD and all causes of mortality [24, 46, 47]. With the 24-h ambulatory blood pressure (ABP) monitoring, the baseline of C-STRIDE showed higher SBP and similar DBP in those with CVD. ABP was recently demonstrated to be more important than office blood pressure for predicting CVD and mortality [46]. Morning surge in blood pressure was shown to be a predictor of stroke in elderly hypertensives [47]. In the CKD-JAC study, where ABP was measured at different times to distinguish the impacts of night and morning blood pressure, a higher morning ABP surge was associated with CVD risk independently [24]. In short, nearly all studies support the importance of effective BP management in CKD as a public health priority.

Internationally, a BMI of 25.0–29.9 kg/m^2^ is considered overweight and a BMI ≥30 kg/m^2^ is considered obese. Based on the BMI data of the Chinese population, the Working Group on Obesity of the International Life Science Institute China Office recommended a BMI of 24 kg/m^2^ as the cut-off value for overweight and 28 kg/m^2^ as the cut-off value for obesity for Chinese [48]. The C-STRIDE cohort and the ROUTE cohort (Japan) [2] had similar BMI, both lower than that in Western studies [3–5]. Although some reports have suggested higher BMI as an independent risk factor for advanced CKD and CVD [49], the link between BMI and CVD is not clear cut. Our study does not support a correlation between higher BMI and CVD. Several studies have shown that higher BMI was actually associated with favorable outcomes. For instance, a BMI greater than 30 kg/m^2^ was associated with lower mortality among 920 patients with advanced CKD in a Swedish study [50]. In the Atherosclerosis Risk in Communities (ARIC) cohort, a higher body size was also associated with better overall survival in stage 3 CKD [51].

Our results demonstrate declining GFR as a major risk factor for CVD prevalence in the C-STRIDE cohort. To better examine the function of eGFR, we employed the staging of 3a and 3b instead of a single stage 3. Although a cohort study of Taiwan found no difference between 3a and 3b in predicting CVD incidence [52], we observed significant differences in the occurrence of MI, cerebrovascular disease and PAD between stages 3a and 3b. A multitude of studies have clearly demonstrated that overt renal dysfunction is independently and significantly associated with an increased risk of CVD events and mortality [53–55]. A study from Japan indicated that even after adjustment for other risk factors, the presence of CKD conferred a higher risk of cardiovascular death with a hazard ratio of 1.20 [53]. A negative graded correlation between eGFR and risk of cardiovascular death was observed. The Framingham Heart Study suggested the same association [54]. The KORA Study demonstrated that CKD was strongly associated with an increased risk of incident MI and CVD mortality, independent from common cardiovascular risk factors in men and women [55]. The MATISS Study suggested that in an elderly general population with low risk of CVD and low incidence of reduced renal function, even a modest eGFR reduction was related to all-cause mortality and CVD incidence [56].

The overall prevalence of AAC in the C-STRIDE study baseline was 32.9%, with statistically higher percentages in stages 3b and 4. Multiple regression analysis indicated that AAC increases the risk for CVD in CKD. Another Chinese study [57] reported an AAC incidence of 54% in the CKD patients, and also showed a strong association between the incidence of AAC and cardiovascular risks. Specifically, AAC was positively correlated with left atrial anteroposterior diameter (LAD), pulmonary arterial systolic pressure (PASP) and carotid artery intima-media thickness (IMT), and negatively correlated with ejection fraction (EF) and shortening fraction (SF) [57]. A cohort study performed on adult Japanese patients with pre-dialysis CKD demonstrated 82% subjects had AAC, and identified AAC as independent predictors for de novo cardiovascular events in CKD stages 4 and 5 [58]. A US study [59] evaluated the association of AAC and CVD in 1974 randomly selected subjects (45 to 84 years old) with complete AAC and coronary artery calcification (CAC) data from computerized tomographic scans. It was found that AAC and CAC predicted hard coronary heart disease and hard CVD events independent of one another. Only AAC was independently related to CVD mortality, and AAC showed a stronger association with total mortality than CAC.

It is worth noting some limitations of our study. First, we had a less-than-anticipated diabetes recruitment, which could cause a potential bias. The strict criteria for DN screening may in part account for the lower diabetes diagnosis in our cohort. The defining eligibility of DN was eGFR 15–59 ml/min/1.73 m^2^, or eGFR ≥ 60 ml/min/1.73 m^2^ with “nephrotic range” proteinuria, which was defined as 24-h urinary protein ≥3.5 g or urinary albumin creatinine ratio (UACR) ≥2 000 mg/g [8]. As a result, early stage DN was not adequately screened for. Nevertheless, this design would ensure sufficient power to observe adverse consequences in the DN-subgroup population, which will provide valuable information on diabetes as a cause of CKD in China. Second, we used self-report and review of medical records to define CVD in this study. This may have missed a small group of participants with undiagnosed CVD, and therefore the results of CVD-related morbidity may not be all-inclusive. Third, abdominal aorta calcification was determined by radiograph, which is less sensitive in detecting atherosclerotic lesions than newer modalities such as computerized tomography [60]. Therefore, early stage vascular calcification may have been under reported. Computerized tomography was not available in this research due to the high costs. However, color Doppler ultrasound has been used in the C-STRIDE cohort to evaluate carotid artery calcification. This will improve diagnostic sensitivity of cardiovascular calcification by integration of radiographic and ultrasound techniques during follow-up.

## Conclusions

In summary, the C-STRIDE baseline analysis has demonstrated that participants with progressive CKD have a higher prevalence of CVD at entry than the general Chinese population. Age, diabetes, hypertension, abdominal aorta calcification and stage 3 & 4 CKD are significantly associated with the prevalence of CVD. In the next phase of the study, all subjects will be sampled annually for at least 5 years. This Long-term follow-up of participants will provide critical insight into the epidemiology of CVD in CKD, reveal the impact of individual risk factors on adverse outcomes, and serve as a foundation for future interventional investigations.

## Additional file

Additional file 1: Table S1. Anticipated and actual target distributions of CKD etiology and renal function, C-STRIDE study (Nov 2011- Mar 2016). (DOC 33 kb)

## Abbreviations

AAC: Abdominal aorta calcification
ABP: Ambulatory blood pressure
ANOVA: Analysis of variance
aOR: Adjusted odds ratios
ARIC: Atherosclerosis risk in communities
BMI: Body mass index
CAC: Coronary artery calcification
CI: Confidence interval
CKD: Chronic kidney disease
CRIC: Chronic renal insufficiency cohort
C-STRIDE: Chinese Cohort Study of Chronic Kidney Disease
CVD: Cardiovascular disease
DBP: Diastolic blood pressure
DM: Diabetes mellitus
DN: Diabetic nephropathy
EF: Ejection fraction
eGFR: Estimated glomerular filtration rate
ESRD: End-stage renal disease
GN: Glomerulonephritis
Hb: Hemoglobin
HbA1c: Hemoglobin A1C
HDL-C: High density lipoprotein cholesterol
hs-CRP: High-sensitivity C-reactive protein
IMT: Intima-media thickness
iPTH: Intact parathyroid hormone
KDIGO: Kidney Disease Improving Global Outcomes
LAD: Left atrial anteroposterior diameter
LDL-C: Low density lipoprotein cholesterol
MI: Myocardial infarction
MOP: Manual of operation procedure
NYHA: New York Heart Association
PAD: Peripheral arterial disease
PASP: Pulmonary arterial systolic pressure
ROUTE: Research and outcome in treatment and epidemiology
SAS: Statistical analysis system
SBP: Systolic blood pressure
SCr: Serum creatinine
SD: Standard deviation
SF: Shortening fraction
TC: Total cholesterol
TG: Triglyceride
UACR: Urinary albumin creatinine ratio
UACR: Urinary albumin creatinine ratio

## References

[1] Zhang L, Wang F, Wang L (2012). Prevalence of chronic kidney disease in China: a cross-sectional survey. Lancet.

[2] Iimori S, Naito S, Noda Y (2015). Anaemia management and mortality risk in newly visiting patients with chronic kidney disease in Japan: The CKD-ROUTE study. Nephrology (Carlton).

[3] Shah R, Matthews GJ, Shah RY (2015). Serum Fractalkine (CX3CL1) and Cardiovascular Outcomes and Diabetes: Findings From the Chronic Renal Insufficiency Cohort (CRIC) Study. Am J Kidney Dis.

[4] Ritchie J, Rainone F, Green D (2013). Extreme Elevations in Blood Pressure and All-Cause Mortality in a Referred CKD Population: Results from the CRISIS Study. Int J Hypertens.

[5] Martinez-Castelao A, Gorriz JL, Portoles JM (2011). Baseline characteristics of patients with chronic kidney disease stage 3 and stage 4 in Spain: the MERENA observational cohort study. BMC Nephrol.

[6] Collins AJ, Foley RN, Chavers B (2012). United States Renal Data System 2011 Annual Data Report: Atlas of chronic kidney disease & end-stage renal disease in the United States. Am J Kidney Dis.

[7] Drury PL, Ting R, Zannino D (2011). Estimated glomerular filtration rate and albuminuria are independent predictors of cardiovascular events and death in type 2 diabetes mellitus: the Fenofibrate Intervention and Event Lowering in Diabetes (FIELD) study. Diabetologia.

[8] Gao B, Zhang L, Wang H, Zhao M (2014). Chinese cohort study of chronic kidney disease: design and methods. Chin Med J (Engl).

[9] Kidney Disease: Improving GlobalOutcomes (KDIGO) CKD Work Group (2013). KDIGO 2012 Clinical Practice Guideline for theEvaluation and Management of Chronic Kidney Disease. Kidney inter.

[10] Levey AS, Stevens LA, Schmid CH (2009). A new equation to estimate glomerular filtration rate. Ann Intern Med.

[11] Lash JP, Go AS, Appel LJ (2009). Chronic Renal Insufficiency Cohort (CRIC) Study: baseline characteristics and associations with kidney function. Clin J Am Soc Nephrol.

[12] Le ML, Wilkens LR, Kolonel LN, Hankin JH, Lyu LC (1997). Associations of sedentary lifestyle, obesity, smoking, alcohol use, and diabetes with the risk of colorectal cancer. Cancer Res.

[13] Feng Z, Liu C, Guan X, Mor V (2012). China’s rapidly aging population creates policy challenges in shaping a viable long-term care system. Health Aff (Millwood).

[14] Yang ZJ, Liu J, Ge JP, Chen L, Zhao ZG, Yang WY (2012). Prevalence of cardiovascular disease risk factor in the Chinese population: the 2007–2008 China National Diabetes and Metabolic Disorders Study. Eur Heart J.

[15] Ohno M, Deguchi F, Izumi K (2014). Correlation between renal function and common risk factors for chronic kidney disease in a healthy middle-aged population: a prospective observational 2-year study. PLoS ONE.

[16] Yamagata K, Ishida K, Sairenchi T (2007). Risk factors for chronic kidney disease in a community-based population: a 10-year follow-up study. Kidney Int.

[17] Go AS, Chertow GM, Fan D, McCulloch CE, Hsu CY (2004). Chronic kidney disease and the risks of death, cardiovascular events, and hospitalization. N Engl J Med.

[18] Ryan TP, Fisher SG, Elder JL (2009). Increased cardiovascular risk associated with reduced kidney function. Am J Nephrol.

[19] Ninomiya T, Kiyohara Y, Kubo M (2005). Chronic kidney disease and cardiovascular disease in a general Japanese population: the Hisayama Study. Kidney Int.

[20] Iimori S, Noda Y, Okado T (2013). Baseline characteristics and prevalence of cardiovascular disease in newly visiting or referred chronic kidney disease patients to nephrology centers in Japan: a prospective cohort study. BMC Nephrol.

[21] Rahman M, Xie D, Feldman HI (2014). Association between chronic kidney disease progression and cardiovascular disease: results from the CRIC Study. Am J Nephrol.

[22] Jin X, Zhou J, Zhou J, Pan X, Chen H, Ge J (2016). Role of novel risk factors in predicting risk of ischemic cardiovascular diseases in middle aged men in twenty years in Shanghai. Zhonghua Liu Xing Bing Xue Za Zhi.

[23] Svensson T, Inoue M, Sawada N (2016). Coping strategies and risk of cardiovascular disease incidence and mortality: the Japan Public Health Center-based prospective Study. Eur Heart J.

[24] Imai E, Matsuo S, Makino H (2010). Chronic Kidney Disease Japan Cohort study: baseline characteristics and factors associated with causative diseases and renal function. Clin Exp Nephrol.

[25] Liu ZH (2013). Nephrology in china. Nat Rev Nephrol.

[26] Xu Y, Wang L, He J (2013). Prevalence and control of diabetes in Chinese adults. JAMA.

[27] Culleton BF, Larson MG, Parfrey PS, Kannel WB, Levy D (2000). Proteinuria as a risk factor for cardiovascular disease and mortality in older people: a prospective study. Am J Med.

[28] Hillege HL, Fidler V, Diercks GF (2002). Urinary albumin excretion predicts cardiovascular and noncardiovascular mortality in general population. Circulation.

[29] Xu J, Knowler WC, Devereux RB (2007). Albuminuria within the “normal” range and risk of cardiovascular disease and death in American Indians: the Strong Heart Study. Am J Kidney Dis.

[30] Wright JT, Bakris G, Greene T (2002). Effect of blood pressure lowering and antihypertensive drug class on progression of hypertensive kidney disease: results from the AASK trial. JAMA.

[31] Brantsma AH, Bakker SJ, Hillege HL, De Zeeuw D, De Jong PE, Gansevoort RT (2008). Cardiovascular and renal outcome in subjects with K/DOQI stage 1–3 chronic kidney disease: the importance of urinary albumin excretion. Nephrol Dial Transplant.

[32] Liao LN, Liu CS, Li CI (2015). Three-year incidence of elevated albuminuria and associated factors in a population-based cohort: The Taichung Community Health Study. Eur J Prev Cardiol.

[33] Fernandez-Fernandez B, Ortiz A, Gomez-Guerrero C, Egido J (2014). Therapeutic approaches to diabetic nephropathy--beyond the RAS. Nat Rev Nephrol.

[34] Molitch ME, Steffes M, Sun W (2010). Development and progression of renal insufficiency with and without albuminuria in adults with type 1 diabetes in the diabetes control and complications trial and the epidemiology of diabetes interventions and complications study. Diabetes Care.

[35] Kramer HJ, Nguyen QD, Curhan G, Hsu CY (2003). Renal insufficiency in the absence of albuminuria and retinopathy among adults with type 2 diabetes mellitus. JAMA.

[36] Retnakaran R, Cull CA, Thorne KI, Adler AI, Holman RR (2006). Risk factors for renal dysfunction in type 2 diabetes: U.K. Prospective Diabetes Study 74. Diabetes.

[37] Garg AX, Kiberd BA, Clark WF, Haynes RB, Clase CM (2002). Albuminuria and renal insufficiency prevalence guides population screening: results from the NHANES III. Kidney Int.

[38] Haller H, Ito S, Izzo JL (2011). Olmesartan for the delay or prevention of microalbuminuria in type 2 diabetes. N Engl J Med.

[39] Mann JF, Schmieder RE, McQueen M (2008). Renal outcomes with telmisartan, ramipril, or both, in people at high vascular risk (the ONTARGET study): a multicentre, randomised, double-blind, controlled trial. Lancet.

[40] Beaglehole R, Yach D (2003). Globalisation and the prevention and control of non-communicable disease: the neglected chronic diseases of adults. Lancet.

[41] Atkins RC (2005). The epidemiology of chronic kidney disease. Kidney Int Suppl.

[42] Levin A, Djurdjev O, Barrett B (2001). Cardiovascular disease in patients with chronic kidney disease: getting to the heart of the matter. Am J Kidney Dis.

[43] Zhang L, Long J, Jiang W (2016). Trends in Chronic Kidney Disease in China. N Engl J Med.

[44] Fernández FB, Elewa U, Sánchez-Niño MD (2012). 2012 update on diabetic kidney disease: the expanding spectrum, novel pathogenic insights and recent clinical trials. Minerva Med.

[45] Wang C, Deng WJ, Gong WY (2015). High prevalence of isolated nocturnal hypertension in Chinese patients with chronic kidney disease. J Am Heart Assoc.

[46] Ohkubo T, Imai Y, Tsuji I (1997). Prediction of mortality by ambulatory blood pressure monitoring versus screening blood pressure measurements: a pilot study in Ohasama. J Hypertens.

[47] Kario K, Pickering TG, Umeda Y (2003). Morning surge in blood pressure as a predictor of silent and clinical cerebrovascular disease in elderly hypertensives: a prospective study. Circulation.

[48] Wu YF, Ma GS, Hu YH (2005). The current prevalence status of body overweight and obesity in China: data from the China National Nutrition and Health Survey. Zhonghua Yu Fang Yi Xue Za Zhi.

[49] Kramer H, Luke A, Bidani A, Cao G, Cooper R, McGee D (2005). Obesity and prevalent and incident CKD: the Hypertension Detection and Follow-Up Program. Am J Kidney Dis.

[50] Evans M, Fryzek JP, Elinder CG (2005). The natural history of chronic renal failure: results from an unselected, population-based, inception cohort in Sweden. Am J Kidney Dis.

[51] Kwan BC, Murtaugh MA, Beddhu S (2007). Associations of body size with metabolic syndrome and mortality in moderate chronic kidney disease. Clin J Am Soc Nephrol.

[52] Chen YC, Su YC, Lee CC, Huang YS, Hwang SJ (2012). Chronic kidney disease itself is a causal risk factor for stroke beyond traditional cardiovascular risk factors: a nationwide cohort study in Taiwan. PLoS ONE.

[53] Nakamura K, Okamura T, Hayakawa T (2006). Chronic kidney disease is a risk factor for cardiovascular death in a community-based population in Japan: NIPPON DATA90. Circ J.

[54] Parikh NI, Hwang SJ, Larson MG, Levy D, Fox CS (2008). Chronic kidney disease as a predictor of cardiovascular disease (from the Framingham Heart Study). Am J Cardiol.

[55] Meisinger C, Doring A, Lowel H (2006). Chronic kidney disease and risk of incident myocardial infarction and all-cause and cardiovascular disease mortality in middle-aged men and women from the general population. Eur Heart J.

[56] Donfrancesco C, Palleschi S, Palmieri L (2013). Estimated glomerular filtration rate, all-cause mortality and cardiovascular diseases incidence in a low risk population: the MATISS study. PLoS ONE.

[57] Bao HD, Sheng XH, Wang NS (2014). Analysis the Occurrence and Risk Factors of Abdominal Aorta Calcification in Advanced CKD Patients. CJITWN.

[58] Hanada S, Ando R, Naito S (2010). Assessment and significance of abdominal aortic calcification in chronic kidney disease. Nephrol Dial Transplant.

[59] Criqui MH, Denenberg JO, McClelland RL (2014). Abdominal aortic calcium, coronary artery calcium, and cardiovascular morbidity and mortality in the Multi-Ethnic Study of Atherosclerosis. Arterioscler Thromb Vasc Biol.

[60] Wilson PW, Kauppila LI, O’Donnell CJ (2001). Abdominal aortic calcific deposits are an important predictor of vascular morbidity and mortality. Circulation.

