# Supplementary material for: Prevalence and risk factors for cardiovascular disease among chronic kidney disease patients: results from the Chinese cohort study of chronic kidney disease (C-STRIDE)
Source: BMC Nephrol. 2017 Jan 14;18:23. doi: 10.1186/s12882-017-0441-9 (PMC5237491; doi:10.1186/s12882-017-0441-9)
Supplement: Additional file 1: Table S1. — Anticipated and actual target distributions of CKD etiology and renal function, C-STRIDE study (Nov 2011- Mar 2016). (DOC 33 kb) [file 12882_2017_441_MOESM1_ESM.doc]

**Supplementary data**

Table S1. Anticipated and actual target distribution of etiology and renal function in the C-STRIDE study.

| Anticipated Distribution  Etiology | Anticipated percentage | Actual number (percentage) |
| --- | --- | --- |
| DN | 30% | 434(13.88%) |
| GN | 30% | 1893(60.56%) |
| Other cause | 40% | 799(25.56%) |
| Missing value |  | 42 |
| eGFR(ml/min/1.73m^2^) |  |  |
| ≧45 | 40-60% | 1472(46.46%) |
| <45 | 40-60% | 1696(53.54%) |

Categorical data are presented as numbers (n) of patients and percentages. DN, diabetic nephropathy; GN,glomerulonephritis.
